# An In Silico Analysis of Troponin I Mutations in Hypertrophic Cardiomyopathy of Indian Origin

**DOI:** 10.1371/journal.pone.0070704

**Published:** 2013-08-13

**Authors:** Gayatri Ramachandran, Manoj Kumar, Deepa Selvi Rani, Venkateshwari Annanthapur, Narasimhan Calambur, Pratibha Nallari, Punit Kaur

**Affiliations:** 1 Department of Genetics, Osmania University, Hyderabad, Andhra Pradesh, India; 2 Centre for Cellular and Molecular Biology, Hyderabad, Andhra Pradesh, India; 3 Institute of Genetics and Hospital for Genetic Diseases, Hyderabad, Andhra Pradesh, India; 4 Department of Cardiology, CARE Hospital, Nampally, Hyderabad, Andhra Pradesh, India; 5 Department of Biophysics, All India Institute of Medical Sciences, New Delhi, India; National Institute for Medical Research, Medical Research Council, London, United Kingdom

## Abstract

Hypertrophic Cardiomyopathy (HCM) is an autosomal dominant disorder of the myocardium which is hypertrophied resulting in arrhythmias and heart failure leading to sudden cardiac death (SCD). Several sarcomeric proteins and modifier genes have been implicated in this disease. Troponin I, being a part of the Troponin complex (troponin I, troponin C, troponin T), is an important gene for sarcomeric function. Four mutations (1 novel) were identified in Indian HCM cases, namely, Pro82Ser, Arg98Gln, Arg141Gln and Arg162Gln in Troponin I protein, which are in functionally significant domains. In order to analyse the effect of the mutations on protein stability and protein-protein interactions within the Troponin complex, an in silico study was carried out. The freely available X-ray crystal structure (**PDB ID: 1JIE**) was used as the template to model the protein followed by loop generation and development of troponin complex for both the troponin I wild type and four mutants (**NCBI ID: PRJNA194382**). The structural study was carried out to determine the effect of mutation on the structural stability and protein-protein interactions between three subunits in the complex. These mutations, especially the arginine to glutamine substitutions were found to result in local perturbations within the troponin complex by creating/removing inter/intra molecular hydrogen bonds with troponin T and troponin C. This has led to a decrease in the protein stability and loss of important interactions between the three subunits. It could have a significant impact on the disease progression when coupled with allelic heterogeneity which was observed in the cases carrying these mutations. However, this can be further confirmed by functional studies on protein levels in the identified cases.

## Introduction

Hypertrophic Cardiomyopathy (HCM), is a disease characterized by thickening of the left ventricle and diagnosed pathologically by myocyte disarray and interstitial fibrosis. Cardiac hypertrophy in HCM is a “compensatory” phenotype due to increased cardiac myocyte stress or altered Ca^2+^ sensitivity of the contractile apparatus imparted by mutations in one of the 8 cardiac sarcomeric proteins or modifier genes [1]. To date, at least 700 mutations have been identified in these genes. The pathogenesis of hypertrophy, a common programmed response of the myocardium to any form of stress, whether caused by a genetic defect or by an acquired condition, involves common pathways. Similarly, pathogenesis of diverse cardiac phenotypes also results from up regulation of expression of a variety of genes in response to the primary impetus provided by the mutant contractile protein [1].

The cardiac myocyte is made of myofibrils and myofilaments. Myofibrils are made up of repeating units called sarcomere which are the basic contractile units of the myocyte. It contains the contractile proteins which are of 2 types- a) thick filament proteins -Myosin heavy chain (MYH7) and cardiac Myosin-binding protein-C (MYBPC3) which is reported to be implicated mostly in HCM b) thin filament regulatory proteins – Tropomyosin (TPM1), cardiac Troponin (cTn) and Actin (ACTC). The myosin has 2 heads with ATPase function which interacts with binding sites on actin forming cross bridges that leads to contraction –relaxation coupling. Tropomyosin is a rod shaped protein that is interdigitated between the alpha helical strands of actin.

Troponin is attached to tropomyosin at regular intervals, and lies within the groove between actin filaments. In cardiac muscle, troponin and tropomyosin form the principal mechanism by which contractility is regulated in response to the Ca^2+^ concentration surrounding the contractile apparatus [2]. Troponin is a component of thin filaments (along with actin and tropomyosin), to which calcium binds to accomplish this regulation. Troponin has three subunits, Troponin C (TnC), Troponin I (TnI), and Troponin T (TnT) and Ca^2+^ sensitivity is conferred on troponin by the troponin C subunit, which is an EF-hand Ca^2+^-binding protein. At micro molar Ca^2+^ concentrations troponin C binds strongly to troponin I, which is central to thin filament regulation, since it is an actin-binding inhibitory protein which on its own inactivates thin filaments and thus prevents it from binding and inhibiting actin. Hence, tropomyosin rolls out of the way of the actin filament active sites, so that myosin can attach to the thin filament and produce force and/or movement. In the absence of calcium, tropomyosin interferes with this action of myosin, and therefore muscles remain relaxed [3]. Troponin T forms the scaffolding of this regulatory switch by binding to troponin C, to troponin I and very strongly to tropomyosin. Regulatory conformational changes in actin, troponin I and troponin C are transmitted efficiently to tropomyosin by troponin T [3].

The integrated nature of the troponin-tropomyosin switch explains how mutations in any component of it can lead to a common outcome [3].

The three subunits of the troponin complex are held together by Ca^2+^-independent interactions between the carboxy terminal domain of Troponin C, the amino terminal domain of Troponin I comprising the first 98 residues and the last 50 residues of Troponin T [4]. The cardiac Troponin I differs from the slow skeletal isoform of Troponin I by a 32 amino acid extension at the amino terminus [5].

Multiple Troponin C and Actin binding domains within Troponin I have been localized to the car boxy region of the protein and this carboxy portion of Troponin I is designated the inhibitory peptide (IP) region which toggles between actin and Troponin C in the absence and presence of Ca^2+^, respectively [6]. The minimum inhibitory peptide region (residues 137–148) of human cardiac Troponin I is rich in basic amino acids, and its sequence is highly conserved [7]. It was also shown that Arg residues in the inhibitory region are involved in the interaction with actin [8].

The amino terminal domain of Troponin I interacts with Troponin T and is involved in the Ca^2+^-independent interactions with Troponin C [7]. Troponin I (1−98) residues interact with 216–263 residues of Troponin T that is essential for the binding of Troponin I-(1−98)–Troponin C to the thin filament; and the residues 1–102 of Troponin I are also required for the binding of Troponin C/Troponin I to Actin-Tropomyosin-Troponin T in the presence of calcium [9].

In a recent study on the Indian population, screening of all the exons including the exon-intron boundaries of the Troponin I gene in 101 HCM patients along with 160 healthy controls revealed a total of 16 mutations, including 15 SNPs, and a 4 bp deletion polymorphism [10]. Of the 16 SNPs, 7 were exonic (one novel, 3 reported non-synonymous and 3 synonymous mutations), and 9 were intronic mutations (Table 1). The Troponin I gene encodes the human cardiac Troponin I. Interestingly, three heterozygous arginine to glutamine (Arg to Gln) substitution at 3 positions 98, 141 and 162 in Troponin I were found. All three arginine → Glutamine mutations at 98, 141 and 162 were exclusively observed in the hypertrophic cardiomyopathy patients. The regions where mutation lies were reported to be the functionally significant domains with Arg141Gln mutation lying in the highly conserved region of the inhibitory peptide of Troponin I. Also, another reported missense mutation Pro82Ser was found in exon 5 of the gene [10].

**Table 1:** List of SNPs/Mutations in Troponin I gene identified in the Indian population.

S.No.	Position	Location	AA Change	Major/Minor Allele	Mutations ObservedControl HCM	Reported/ Novel
1	g.1389	Intron 1	----	(T/C)	Nil 2	rs11667847
2	g.1403	Intron 1	----	(A/G)	Nil 1	rs11671293
3	g.1215	Intron 1	----	(C/A)	Nil 1	rs 3729707
4	g.1486-90	Intron 1	4bp del/In	ACAG	P P	Polymorphic
5	g.1698	Intron 2	----	(T/C)	Nil 4	rs 3729836
6	g.1810	Intron 3	----	(G/A)	Nil 3	rs 3729837
7	g.1897	Intron 3	----	(G/A)	Nil 22	rs 3729838
8	g.2560	Exon 5	R68R	(G/T)	Nil 4	rs 3729711
9	g.2563	Exon 5	R69R	(C/A)	Nil 2	Reported
10	g.2601	Exon 5	P82S	(C/G)	Nil 1	Reported
11	g.2653	Intron 5	----	(G/A)	Nil 1	Novel
12	g.4003	Intron 6	----	(C/T)	Nil 1	Novel
13	g.4019	Exon 6	R98Q	(G/A)	Nil 1	Novel
14	g.4682	Exon 7	R141Q	(G/A)	Nil 1	Reported to be associated with HCM
15	g.4745	Exon 7	R162Q	(G/A)	Nil 17	Reported to be associated with HCM
16	g.4797	Exon 7	E179E	(G/A)	Nil 17	rs 3729841

The high prevalence of Arginine → Glutamine mutations (4%) at three positions (Arg98Gln, Arg141Gln, Arg162Gln) and the absence of remaining reported mutations observed in other populations (2–7%), clearly elucidates the unique origin of Indian populations [10].

Hence, it was found fit to evaluate the influence of the mutations (Pro82Ser, Arg98Gln, Arg141Gln and Arg162Gln) in the disease progression by predicting the molecular changes in the protein through in silico models generated with the help of standard bioinformatics tools. This study is the first of its kind, considering that there is no available literature yet on the in silico modelling of the cardiac protein Troponin I and its mutant analogues, with reference to Indian population.

These mutations could be predicted to significantly affect the disease progression due to protein instability in combination with allelic heterogeneity.

## Results

**Figure 1:**
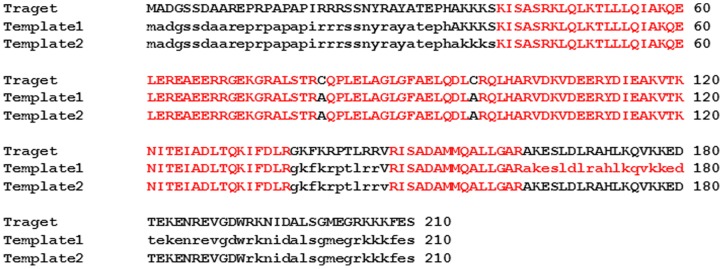
Sequence Alignment. Target protein, human Troponin I, and templates (PDB ID: 1J1E) were aligned using BLAST (template 1: Chain C and template 2: Chain F). The identical residues with available co-ordinates from both the templates are in red and the corresponding residues without available co-ordinates are indicated as lower case.

**Figure 2:**
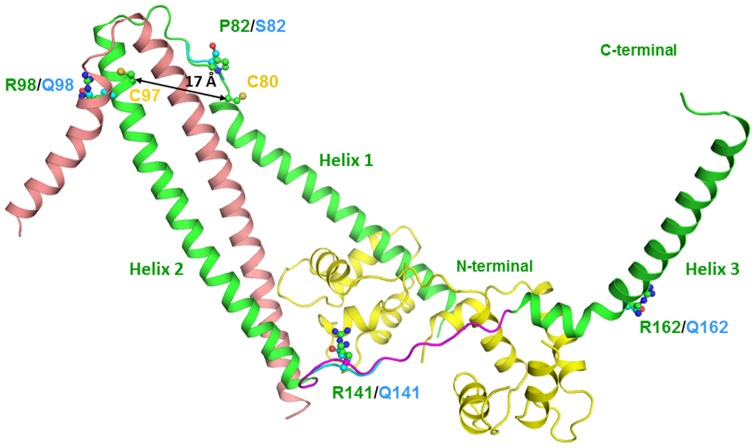
3-D Structure of troponin complex (cartoon). Figure shows TnI (green), TnC (wheat) and TnT (yellow) and the superimposed structures of the various mutants of mutant TnI (cyan).The side chain residues at mutation site in TnI in both native (green) and mutants (cyan) are drawn. The generated loop in model structure (magenta) and the two cysteines (yellow) are indicated.

The sequences of target and template proteins were identical except for two residues (alanine) at positions 80 and 97 which is cysteine in the present protein (Fig. 1). Though these residues were modelled as cysteine they do not form a disulphide linkage as they are 17Å apart. The cysteine residue at 97^th^ position is adjacent to the site of mutation, Arg98Glu but does not influence the side conformation of the neighbouring residues. The main chain conformations of all the non-glycine residues in the model structure were found to be in the allowed regions of Ramachandran plot. The model structure consists of three long helices connected by loops similar to the template structure. The model structure generated was continuous for the region comprising residues 40 to 191 wherein the helix 2 and helix 3 are connected by a loop (residues 137 to 146). This loop was not observed in the template structure (Fig. 2).

### Loop Generation

The co-ordinates for the residues 137–146 were generated by loop modelling program and resulted in 3-D structure of continuous region of Troponin I protein from residues 40–191. The region of residues from 137 to 146 was modelled as loop (Fig. 2). Hence, the developed structure of Troponin I contained co-ordinates from X-ray diffraction data for residues 40–136 and 147–191 as well as co-ordinates generated by modelling program from residue 137–146 including the side chain atoms of two cysteine residues. The main chain conformation of 9 of the 10 residues in the modelled region were found to be in the most favoured regions of Ramachandran's plot while residue Phe139 in additionally allowed region. None of the residue in the modelled region lies in the disallowed region of Ramachandran's map. This verified the good quality of modelled region. The overall structure consisted of three long helices connected by two loops. The modelled loop (residues 137 to 146) connects helix 2 and helix 3. This structure was used to develop the model structure of mutant Troponin I protein. The structure of the native as well as mutant Troponin I including the modelled region was used to develop the structure of Troponin complex (Fig. 2) to study the effect of point mutation on the interaction between the three subunits of Troponin complex. The modelled structure of wild type troponin I with *de novo* modelled loop of 10 residues and model structure of mutants was submitted in BioProject at **NCBI** with accession ID of **PRJNA194382**.

### Arg98Gln

**Figure 3:**
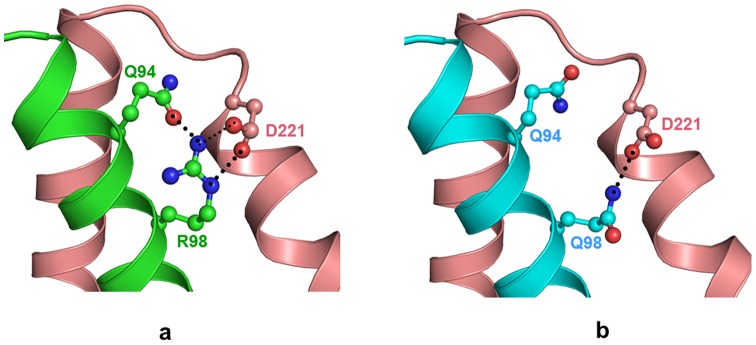
R98Q mutation reduces intermolecular contacts with Troponin C protein. Model structure of (a) wild type troponin complex, TnI (green) and TnC (wheat) (b) mutant structure TnI (cyan). The side chain of Arg98 and its interacting residues are shown (ball and stick) in respective color and hydrogen bond interactions are indicated as black dotted lines.

**Figure 4:**
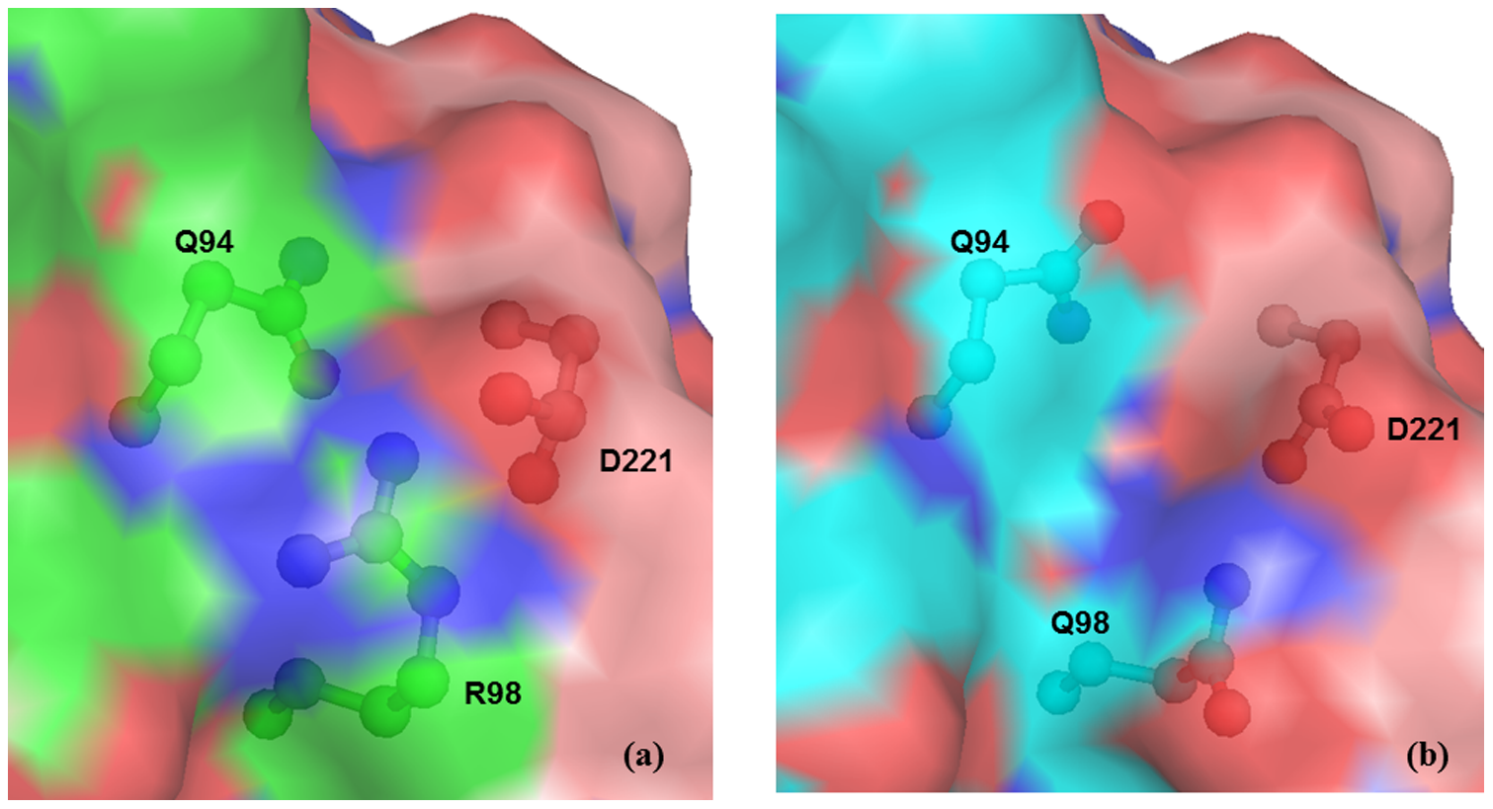
Surface diagram of wild type and mutant (Arg98Gln) Troponin complex. (a) model structure of wild type troponin complex, TnI (green) and TnC (wheat) and (b) model structure of mutant (Arg98Gln) TnI (cyan) in troponin complex indicating the side chain of Arg98/Gln98 and interacting residues (ball and stick). Surface potentials are different in wild type and mutant due to the difference of side chain as well as due to flipping of amide group in Gln94.

The change at residue 98 from Arg to Gln due to point mutation does not affect the overall conformation of the protein. The point mutation occurs in Helix 2 but does not disturb the stability of the α-helical secondary structure. However, the two residues, Arg and Gln, differ in both size and charge as Arg is larger and more positively charged compared to the smaller Gln. The Arg98 in wild type Troponin I forms a intramolecular hydrogen bond with side chain of Gln94 of Troponin I and two intermolecular hydrogen bonds with OD1 and OD2 of Asp221 of Troponin C protein (Fig. 3a). This facilitates specific and strong intermolecular interactions with Troponin C protein in the complex. In the case of the mutant, the shorter Gln98 can easily be accommodated but is incapable of forming intramolecular hydrogen bond due to its reduced size (Fig. 3b). However, it forms a intermolecular hydrogen bond with OD1 of Asp221 of TroponinC protein. This leads to a reorientation of the side chains of residues Gln94 of Troponin I and Asp221 of Troponin C subunit. The amide group of Gln98 flip by approximately 180° (Fig. 4). The electrostatic attraction between positive charge on guanidium group of Arg98 in Troponin I and negative charge on Asp222 of Troponin C is also reduced because of the neutral nature of Gln98 in the mutant Troponin I. The electrostatic attraction with negative charge on Asp222 of Troponin C is also reduced due to the comparatively neutral nature of mutant Gln98. Hence the change in nature and size of residues due to mutation Arg98Gln in Troponin I protein significantly reduces the protein-protein interactions with Troponin C in the troponin complex. The calculated potential energy of the wild type protein is −3313 kcal/ mol compared to −3220 kcal/mol for mutant protein. This may be due to the loss of intramolecular hydrogen bonds between side chain of Gln98 and neighbouring residues. Moreover, the region comprising residue 98 is surface exposed and has been indicated to be the binding site for protein Troponin C.

### Arg141Gln

**Figure 5:**
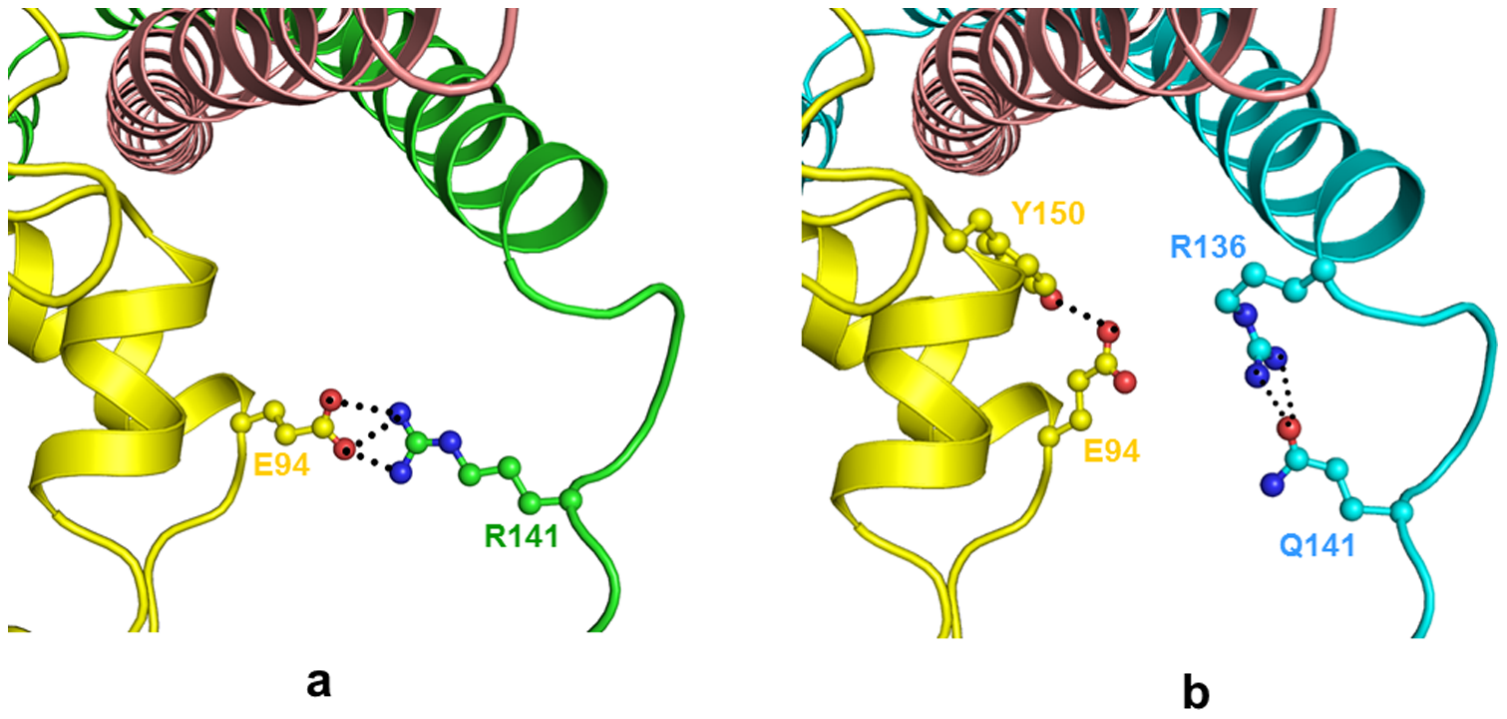
Arg141Gln mutation. Model structure of troponin complex (cartoon) of (a) wild type, TnI (green), TnT (yellow) and TnC (wheat) and (b) mutant (Arg141Gln) TnI (cyan). The side chain of Arg141/Gln141 and interacting residues (ball and stick) are in respective color. The hydrogen bond interactions are shown as black dotted lines.

The residue Arg141 in Troponin I occurs in the loop region. This basic Arg residue in the mutant is replaced by the shorter Gln residue. The Arg141Gln point mutation produces a minor local conformational change due to the difference in the observed contacts, resulting in an increase in the potential energy (−3238 kcal/mol) (Table 2). The guanidium group of Arg141 in Troponin I is involved in the formation of intermolecular hydrogen bonds with Glu94 side chain of Troponin T subunit (Fig. 5a). The shorter and more hydrophilic Gln in the mutant is unable to retain this contact and in contrast is involved in intra chain hydrogen bonded interaction (Fig. 5b).

**Table 2:** Potential energy of wild type and mutant troponin I.

Troponin I	Potential Energy (kcal/mol)
Wild Type	−3313
Arg98Gln	−3220
Arg141Gln	−3238
Arg162Gln	−3231
Pro82Ser	−3321

### Arg162Gln

**Figure 6:**
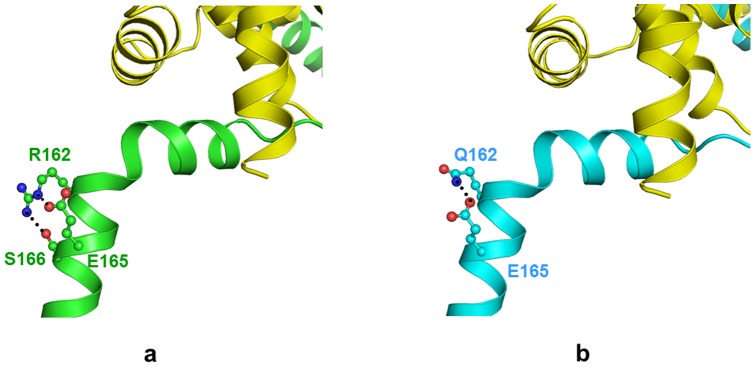
Arg162Gln mutation. Model structure of troponin complex (a) wild type TnI (green) and TnT (yellow) and (b) mutant TnI (cyan). The side chain of Arg162/Gln162 and interacting residues (ball and stick) in respective color. The hydrogen bond interactions are black dotted lines.

The 162 residue present in Helix 3 of Troponin I is positioned away from the region of contact between the three subunits of troponin complex. Thus the substitution of this residue may not affect the protein-protein interaction, but the residue Arg162 forms intra chain hydrogen bonds with carboxylate oxygen of Glu165 and hydroxyl group of Ser166 (Fig. 6a). Loss of intra-chain contacts occurs as the shorter Gln162 in mutant Troponin I can retain only the hydrogen bonded contact with Glu165 (Fig. 6b). Hence, the mutant is less stable which is supported by a decrease in (less negative) in potential energy (−3231 kcal/mol) as compared to wild type troponin I (−3313 kcal/mol) (Table 2).

### Pro82Ser

**Figure 7:**
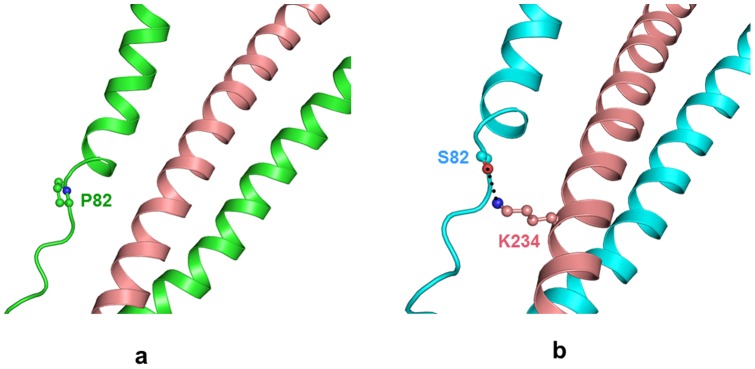
Pro82Ser mutation. Troponin complex model structure of (a) wild type TnI (green) and TnC (wheat). Pro82 forms no interactions (b) mutant TnI (cyan). Ser82 interacts with Lys234 from TnC. The residues (ball and stick in respective color) with hydrogen bond (black dotted lines) are indicated.

In the native structure, the Proline residue, located in the loop region, is not involved in any intra or intermolecular interactions. Proline possesses an imino group and the change to Serine introduces a polar hydroxyl group which unlike Proline has hydrogen bonding capabilities. But hydroxyl group of Ser82 side chain in mutated protein does not form any inter molecular hydrogen bond and hence potential energy does not change much (−3321 kcal/mol) (Table 2). However, this substitution results in hydrogen bonded interaction with the butyl ammonium nitrogen atom of Lys234 in the Troponin C subunit of troponin complex (Fig. 7). As a consequence of this additional contact, a slight local perturbation of the loop conformation in the protein is observed.

## Discussion

This study mainly highlights on arginine to glutamine substitution mutations in Troponin I which are found to be highly prevalent in the Indian population leading to sudden cardiac death (SCD) [10]. Arginine generally prefers to be on the surface of the protein, but its amphipathic nature can mean that part of the side chain is buried. Arginines are also frequently involved in salt-bridges and contains a complex guanidinium group on its side chain that has a geometry and charge distribution that is ideal for binding negatively-charged groups (like negatively-charged non-protein atoms) where they pair with a negatively charged aspartate or glutamate to create stabilizing hydrogen bonds that can be important for protein stability explaining the high incidence of arginine to glutamine substitutions. Arginine/Glutamine substitutions are quite frequent in protein active or binding sites which explains why it is important to study their mutational effects in HCM associated with SCD [11].

These mutations mainly show the effect of hydrogen bonds on the stability of protein as well as stability of intermolecular assembly. In the unfolded state, all potential hydrogen bonding partners in the extended polypeptide chain are satisfied by hydrogen bonds to water. When the protein folds, these protein-to-water hydrogen bonds are broken and only some are replaced by sub-optimal intra-protein hydrogen bonds. Despite the small contribution made to protein stability by hydrogen bonds, if an intramolecular hydrogen bond in a protein is disrupted, without the possibility of forming a compensating hydrogen bond to solvent, that protein will be destabilized [12].

The novel mutation Arg98Gln in exon 6 of Troponin I gene, was observed in a 28 years old severe hypertrophic obstructive cardiomyopathy patient (HOCM) with interventricular septum (IVS) thickness of 25 mm and his 5 years old asymptomatic son, who has a family history of sudden cardiac death [10]. The change in nature and size of residues due to mutation Arg98Gln in TnI protein alters its structural properties in terms of both intra- and inter-molecular contacts with TnC. Moreover, the wild type protein seems to be more stable as compared to mutant protein (Arg98Gln) based on energy parameters (potential energy of the wild type protein is −3313 kcal/mol in comparison to −3220 kcal/mol for mutant protein), as a result the mutant protein is predicted to be destabilised and degraded in the cardiomyocyte (Table 2). This further destabilises the protein-protein interaction of the troponin complex which is essential for relaxation/contraction of the heart muscle.

The dominant Arg141Gln mutation in exon 7 of Troponin I gene was observed in two individuals with severe familial asymmetric septal hypertrophy (ASH+), with interventricular septum (IVS) thickness of 25 and 28 mm, respectively [10]. This substitution disrupts the stabilizing intermolecular contacts existing between the two subunits Troponin I and Troponin T of troponin complex. This mutation also decreases the stability of Troponin I protein due to entropic cost. The calculated potential energy of Arg141Gln mutant protein (−3238 kcal/mol) is lower than the wild type (−3313 kcal/mol) indicating that the mutated protein is less stable (Table 2).

The dominant Arg162Gln mutation in exon 7 of Troponin I gene was observed in an individual with severe asymmetric septal hypertrophy (ASH) with mean thickness of 29 mm had abnormal echocardiogram/ECG [10]. Both these mutations (Arg141Gln, Arg162Gln) are located in the carboxy terminal part of troponin. Screening for this mutation (Arg162Gln) in all the available family members revealed its presence in 9 individuals [10]. Seven out of 9 individuals with Arg162Gln mutation showed allelic heterogeneity having a synonymous mutation at g.4797: G → A: Glu179Glu in exon 7, which replaces a very frequent codon (GAG: 85%) with rare codon (GAA: 14%). History of sudden cardiac death was also been recorded in this family [10]. Most Mendelian monogenic “loss of function” diseases display inter-individual variability in phenotypic outcome; parameters such as age of onset and severity and range of symptom [13], [14], [15].

It is known that the non-random use of synonymous codons creates codon usage bias [16], affecting the translational speed and co-translational folding and protein structure [17], [18]. Hence, the significance of synonymous codon still being debatable, the 7 out of 9 individuals, who showed allelic heterogeneity (Arg162Gln and Glu172Glu), four were severely affected in the family therefore the implication of a second synonymous mutation (Glu172Glu) cannot be ruled out with the pathogenesis.

The mutant does not introduce any conformational variation as it is housed in the region of the stable helical secondary structure but results in a reduction in the protein stability as indicated by lesser negative potential energy (−3231 kcal/mol) compared to wild type. This region of the protein does not make direct contact with other subunits of the complex and hence the allelic heterogeneity may be involved in the disease progression which is supported by the fact that four out of 7 Individuals with the allelic heterogeneity had presented with severe septal hypertrophy (ASH++) with the mean thickness of 27, 28, 29, 32 mm and the ECGs were abnormal in all the four individuals [10].

C → T transition resulting in the replacement of proline with serine (Pro82Ser) in exon 5, lying within the troponin C binding domain, was observed in 2 HCM patients and a control individual. This mutation improves the protein-protein interactions between the TnI and TnC subunit due to an additional hydrogen bond being formed.

Destabilisation leads to rapid degradation of the mutant protein by cellular proteases, which reduces the steady-state concentration of the protein within the cell. The susceptibility of a protein to such proteolytic breakdown depends upon its kinetics of monomer folding and oligomer assembly and upon the intrinsic (thermodynamic) stability of its functional native-state conformation [19]. Other cellular proteins, notably molecular chaperones, promote correct protein folding and assembly and thus provide some protection against degradation. Recent evidence indicates that premature or accelerated degradation of mutant proteins, provoked by aberrations in their conformation, occurs in various subcellular compartments and represents a significant and prevalent pathogenic mechanism underlying genetic diseases. Inter-individual variability in proteolytic and folding systems can in part explain why “simple monogenic diseases” often display inconsistent genotype-phenotype correlations revealing the complexity of the disorder. Mutations can decrease the thermodynamic stability (i.e. increase the free energy) of the functional native state conformation (Table 2). Such reduced stability increases the propensity of the protein to unfold from the native state [19].

Hence three of the four mutations destabilise the protein while the Pro82Ser mutation stabilises the protein complex.

## Conclusions

The effect of 4 exonic mutations (1 novel) of troponin I which were identified in the Indian population, were analysed by *in silico* protein modelling for the first time. It was hypothesized that these mutations (especially, the Arg to Gln substitutions) may be responsible for gross protein instability which in turn might affect its interactions with other sarcomeric proteins, hence leading to the pathogenesis of cardiac hypertrophy in the HCM patients. The hypothesis proved correct since it was found that these mutations lead to local perturbations in the protein making the complex less stable and it was observed that allelic heterogeneity may also be involved in the disease progression.

The conformational changes induced in the protein structure due to the various point mutations were analysed *in silico* in this study. Further functional studies need to be done to establish protein turnover in wild type and mutant cell lines in order to validate the hypothesis that the protein instability induced due to the mutations and allelic heterogeneity are responsible for the disease progression and severity.

## Methods

The effect of the mutations (Pro82Ser, Arg98Gln, Arg141Gln and Arg162Gln) on the structure and function of the Troponin I protein was analysed. The X-ray crystal structures of human cardiac Troponin I protein in complex with Troponin C and Troponin T (**PDB id: 1J1E**) was available [20]. The Troponin I protein used as the template comprises the chains C and F of the troponin complex. The structures of both the chains together consist of two fragments corresponding to regions from residues 35 to 136 and residues 147 to 191. The co-ordinates of linking region from residues 137 to 146 are not available. These 10 missing atomic co-ordinates for the target protein model structure were generated using the loop modelling program of MODELER 8.2 [21], [22] available in Discovery Studio (DS) 2.0 [23]. The Modeller protocol develops model structure of protein based on homology derived restraints of dihedral angles and distance for main chain and side chain atoms. However, region to be modelled without co-ordinates in reference/template structure is developed by loop modelling algorithm integrated in the Modeller 8.2 protocol. The loop modelling program of Modeller 8.2 uses an energy function optimization based approach. It is based on energy minimization using conjugate gradient algorithm [24] and molecular dynamics with simulated annealing method [25]. The generated loop was further refined by loop refinement program of Modeller 8.2 [26]. This loop refinement program is based on a pseudo-energy function called DOPE (Discrete Optimized Protein Energy) [26]. DOPE is a statistical potential calculated from sample of protein structures in database and dependent on atomic distance. The conformation of the side chains of the residues in the modelled region of the Troponin I structure after loop refinement was subjected to side chain refinement program – ChiRotor [27]. This molecular modelling algorithm performs the refinement by systematic search of side chain conformation followed by energy minimization. The developed structure of Troponin I include the co-ordinates from crystal structure for residues 40–136 and 147–191 and modelled region for residues 137–146. The modelled region of Troponin I protein from residues 137–146 was further energy minimized by conjugate gradient energy minimization programs with the help of molecular mechanics force field parameters of CHARMM (c33b1) [28], [29] and convergence criteria of 0.05 kcal/mol/Å to eliminate any strain present in the structure. The stereochemical quality of model region was assessed by Verify3D [30] and ProCheck programs [31].

The structure for each mutant (Pro82Ser, Arg98Gln, Arg141Gln and Arg162Gln) of Troponin I was developed individually using structure developed for native protein along with newly generated loop with the help of ‘Build Mutant’ protocol of DS 2.0. This protocol changes the selected residue into the desired residue and optimizes the conformation of mutated residue along with neighbouring residues using MODELER. The modelled region of the mutant structure was refined individually by subjecting to similar energy minimization protocol that was used for the native structure. The potential energy of native troponin I and four mutants was calculated using ‘calculate energy’ protocol available in DS 2.0 in the presence of distance dependent dielectric constant and spherical cut-off model of electrostatics with the help of CHARMm force field (c33b1).

The crystal structure of Troponin I protein complexed with Troponin C and Troponin T was used as framework to develop the complex structure of Troponin that includes the modelled region of Troponin I (137–146). The structure of native as well as mutant Troponin I along with modelled region (137–146) was superimposed on the chain corresponding to Troponin I of crystal complex. This newly developed complex was refined by three steps of energy minimization to relax the constraints on the geometry in the newly built structure of the complex. The energy minimization was performed using the force field parameters of CHARMm (c33b1) [28], [29] in the presence of implicit solvent model of distance dependent dielectric using three different minimization algorithms. First step minimization was performed to remove the short contacts using steepest descent algorithm [32] with the convergence criteria of r.m.s (root mean square) gradient of <0.1 kcal/mol/A with the rigid main chain. This was followed by minimization using conjugate gradient algorithm [24] with the convergence criteria of r.m.s gradient of <0.05 kcal/mol/A to relax the complex keeping main chain of the protein restrained. In the final step of minimization, the resultant model was fully minimized using Adopted Basis-set Newton-Raphson (ABNR) algorithm [28] with the convergence criteria of r.m.s gradient of <0.01 kcal/mol/A without any restraint. Similarly troponin complex was developed and refined using the mutant structure of troponin I. These refined complexes were used to study the effect of mutations in Troponin I on overall complex structure of Troponin.
